# Characterization and validation of an in vivo confocal Raman spectroscopy led tri‐method approach in the evaluation of the lip barrier

**DOI:** 10.1111/srt.12814

**Published:** 2019-12-09

**Authors:** Stephan Bielfeldt, Sabrina Laing, Tomasz Sadowski, Hemali Gunt, Klaus‐Peter Wilhelm

**Affiliations:** ^1^ proDERM Institute of Applied Dermatological Research GmbH Hamburg Germany; ^2^ Burts Bees Durham NC USA

**Keywords:** closed chamber transepidermal water loss, lip skin measurements, lip stratum corneum, Raman spectroscopy, skin hydration capacitance

## Abstract

**Background/Aim:**

It was the aim to establish and validate in vivo confocal Raman spectroscopy for characterization of the lip barrier in conjunction with transepidermal water loss (TEWL) and skin capacitance assessments. For the first time in vivo, barrier‐relevant components of the lip (derived, natural moisturizing factors (NMFs) and ceramides are described.

**Methods:**

In 32 healthy volunteers, a dental tongue fixation device was inserted to prevent both voluntary and involuntary lip moisturization during measurements. Seventeen individual parameters relating to water, ceramide, and NMF content were assessed via Raman spectroscopy. Additionally, corneometry and TEWL were measured. To give a guidance for the required volunteer group size of future lip barrier studies for all test parameters, coefficients of variation (CV) were calculated and plots showing the required sample size for a given percentage treatment effect.

**Results:**

Raman spectroscopy assessed parameters on the lower lip comprehensively characterized the state of the lip barrier. Parameter variability was sufficiently low to corroborate changes in most parameters using relatively small study populations.

**Conclusions:**

Lip skin is comparatively well hydrated. Biophysical measurement of the lip barrier function is a challenge, as unconscious licking of the lower lip has to be prevented. In vivo confocal Raman spectroscopy provides insightful parameters for the characterization of the lip barrier and sufficiently low inter‐individual variability to assess relatively small parameter changes employing relatively few study subjects. Differences at the molecular level and at a high spatial resolution are detectable, and these insights might provide a breakthrough in the evaluation of lip barrier function and developing solutions for lip care.

## 1 INTRODUCTION

The lips (labia oris) form an extension from the mucosal membrane to the outer skin. These vermilion borders are the only part of the facial anatomy where the oral mucosa is permanently exposed to the environment.^1^ As such they are highly susceptible to environmental exposure, such as wind, sun, smoking, and temperature extremes. Their prominent presence on the face makes them vulnerable to various diseases and a key target for cosmetic and pharmaceutical treatments. Environmental damage as well as certain medications can cause the lips to become dry, chapped, and less bright in color.^2^ The main cause of dryness and chapping is considered a result of low stratum corneum (SC) moisture capacity and impaired barrier function.^3^, ^4^ It appears that incomplete corneocyte formation of the lip surface is responsible for poor barrier function and water‐holding capacity.^5^ It has been reported that the upper lip is more hydrated than the lower notwithstanding a lack of correlation between lip capacitance and clinical scores of lip dryness.^6^

Lips are covered by a thin layer of stratum corneum with their red coloration believed to result from a combination of decreased density of keratin and translucency of the tissue allowing visualization of the underlying capillaries.^7^, ^8^ Age‐related changes to the lips and perioral skin show that wrinkle number and visibility are linearly related to age, becoming more visible during the fourth and fifth decades. Histological analysis of the upper lip reveal that elastic and collagen fibers in the cutis undergo a degeneration process during the aging process with thinning of the cutis.^9^ The intercommissural distance increases with age, whereas lip height decreases.

A number of studies have used corneometry and transepidermal water loss (TEWL) as methods for examining the hydration and dryness of lips.^10^, ^11^ While it is well known that SC plays an important role in the barrier and water‐holding functions of the skin, ceramides (CERs) are the important SC lipids for maintaining SC functions. The relationship between lip roughness and ceramide profiles has been reported, suggesting that not only the level of total CERs but also the specific CER species and their carbon numbers affect the maintenance of SC function of the lips.^10^ Furthermore, although xerosis represents a physiological response of the SC to environmental threats, the influence of the environmental dew point (DP) is not fully understood in terms of its relationship with the water‐holding capacity of the lips and their environment. Utilizing a dermal phase meter, one study showed that SC water‐holding capacity was discretely influenced by DP.^12^

As a sound investigative and widely accepted method,^13^, ^14^, ^15^ in vivo confocal Raman spectroscopy (CRS), is often employed to study the composition of the epidermal barrier in a space‐resolved manner.^16^ Most of the important body and face regions can be investigated with this device including the lips. However, to our knowledge no in vivo studies on lip skin have been published using confocal Raman spectroscopy. It is a sensitive method that enables biochemical information about the state of skin tissue, while maintaining the capability of delivering this information real time, in an automated non‐invasive manner. By employing this method, a semiquantitative analysis of skin barrier components can be analyzed, for example, SC lipids and natural moisturizing factors, CRS has emerged for high spatial and temporal resolution evaluation of SC barrier function and hydration.^17^, ^18^, ^19^ It permits the direct measurement of SC molecular composition and distribution by combining the principle of a confocal signal acquisition with inelastic (Raman) photon scattering. The signal, coming from a small and spatially defined volume of tissue, can be defined as an “optical sectioning” of the skin.

For further understanding, we wanted to investigate Raman parameters of spatially resolved lip water content, NMF, and ceramide content in comparison with established advanced in vivo methods for evaluating and characterizing both stratum corneum capacitance and transepidermal water loss (TEWL) of the lip skin. Our intention was to establish consistency and validity of these combined methods for meaningful lip product(s) evaluation. Lip treatment products often incorporate oils, waxes, and paraffins in their formulations, with the desired outcome of preventing skin barrier dryness and roughness as well repairing this condition. Well‐established capacitance methods measure the degree of hydration including the widely used Corneometer.^20^ This skin surface measurement utilizes electrical capacitance to gain insight into the water‐holding capacity of the upper layers of the skin. An increase in capacitance values demonstrates a moisturizing effect.^21^

On the other hand, TEWL is often used to measure “moisturization,” by the degree which water evaporates from skin.^22^ TEWL is well‐recognized as the main indicator of skin barrier function.^23^ The open‐chamber method of measurement has established itself as the main method for TEWL measurement. However, it has a number of limitations, especially disturbance by ambient air movements and volunteers breathing (when used on the face). This important limitation was overcome by closing the measurement chamber.^24^

## 2 MATERIAL AND METHODS

This non‐invasive study was executed according to the principles of good clinical practice (GCP), with healthy volunteers providing their written informed consent for inclusion.

Thirty‐four healthy female volunteers were recruited with a mean age of 48.4 ± 13.0 years (mean ± standard deviation). Measurements of ceramide content and water profiles were performed by in vivo confocal Raman spectrometer (Model 3510 Skin Analyzer; RiverD) on a sub‐panel of 23 of the recruited subjects. Skin moisturization was measured by Corneometer^®^ (CM 825; Courage & Khazaka), and transepidermal water loss (TEWL) was measured by Aquaflux^®^ (AF200, Biox Systems Ltd.) on 32 subjects providing valid data.

### 2.1 Study inclusion criteria

Inclusion required volunteers willing to actively participate in the study and abide by its requirements. Written informed consent was required for participation. The study was open to female participants aged 18‐65 years with a BMI of <30 and Fitzpatrick skin types I‐III with uniform skin color and absence of erythema or dark pigmentation within the test area.

The volunteers were required to have normal to dry lips of average size (particularly the lower lip).

### 2.2 Study exclusion criteria

Exclusion criteria required were pregnancy or lactation, drug/alcohol disorders, and infectious diseases; cancer and those undergoing chemotherapy and/or radiotherapy; topical medication at the test site within 7 days prior to study commencement; history of any acute or chronic disease that could interfere with or increase the risk on study participation; active (flaring) disease or chronic skin allergies (atopic dermatitis/eczema), or recently treated skin cancer (within the last 12 months); visually dry, chapped, fissured, or cracked lips at the baseline visit; planned surgeries and/or invasive medical procedures during the course of the study; medical procedures, such as laser resurfacing, or plastic surgery to the test sites (lips) within the last 2 months; history of hypersensitivity to any cosmetics and/or personal care products; history of immunosuppression/immune deficiency disorders (including [HIV infection or AIDS]) or currently using immunosuppressive medications; sunburn, abrasions, scar tissue, tattoos, or diseases of the skin that might interfere with the evaluation made in this study, or that expose study participants to unacceptable risks as determined by a technician; uncontrolled diseases such as asthma, diabetes, hypertension, hyperthyroidism, or hypothyroidism regular use of tanning beds.

## 3 Measurements

### 3.1 Climatic conditions

**Figure 1:**
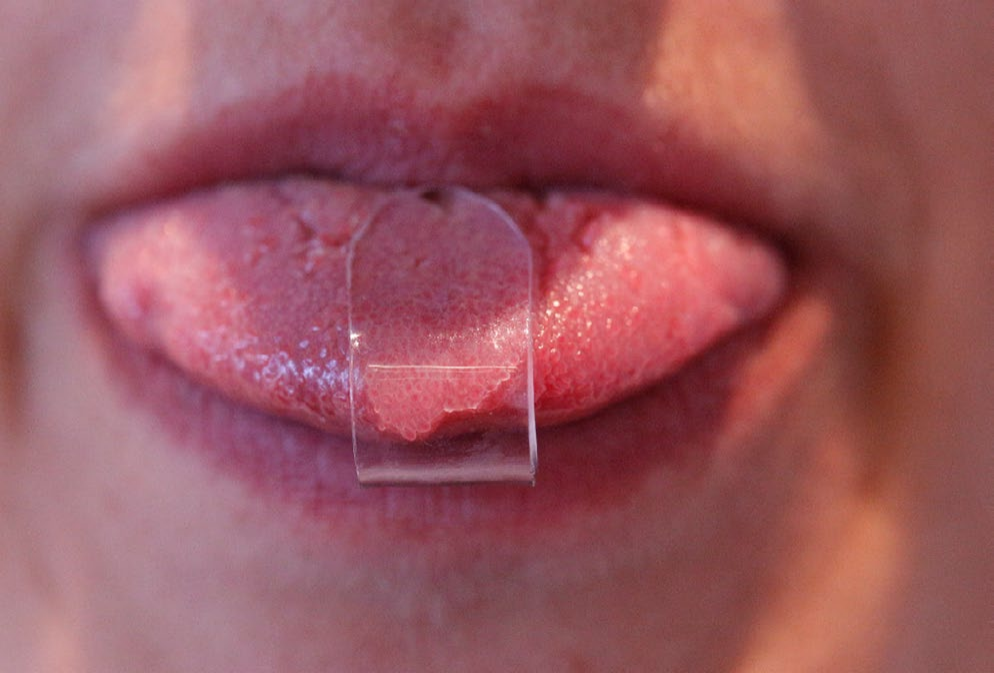
Picture of a dental plastic device to fix the subject's tongue

Instrumental measurements took place in an air‐conditioned room at a temperature of 21 ± 1°C and at 50 ± 5% relative humidity. Subjects remained climatized for at least 30 minutes beforehand. To avoid undesirable moisture accumulation of the lower lip during acclimatization and measurements, a dental plastic device was fixed to the subject's tongue (Figure 1). The tongue device is not inhibiting the ability to move the tongue. It functions as a reminder and in that manner inhibits unconscious licking.

### 3.2 Raman measurement(s)

Raman spectra were obtained by focusing low power laser light in the skin and measuring the Raman scattered light from the laser focus with a confocal Raman Spectrometer Model 3510 Skin Analyzer (River Diagnostics, Rotterdam, Netherlands). A small amount of the scattered light is found at wavelengths higher than the incident laser light. This part of the scattered light provides information about the molecular composition of the skin. Approximately 8 profiles per test area and assessment time were captured in the center part of the lower lip.

#### 3.2.1 Water profile

**Figure 2:**
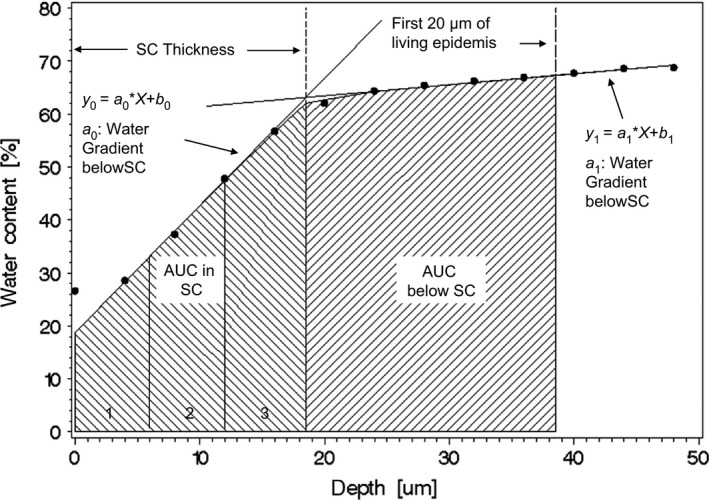
Scheme for Raman water curve with the assessed parameters

The concentration profiles (approximately 8) were calculated from Raman spectra (wavenumber ranged from 2600 to 3800 cm‐1) that were taken at different depths. Profiles were defined by water content at skin depths of 0, 2, 4, 6, up to 24 μm, measured from the surface of the skin, in steps of 2 μm. The following parameters were assessed from obtained water profiles: water content at a depth of 0 μm; water content within stratum corneum including stratum disjunctum (water content in total and water content in three equally divided parts); water contents below the stratum corneum (water content in deeper layers); water gradient within and below the stratum corneum; and thickness of stratum corneum including stratum disjunctum (Figure 2).

#### 3.2.2 Total ceramide profiles

The concentration profiles of total component (ceramide [a.u.]) and total NMF (Total NMF [a.u.]) were calculated from Raman spectra (wavenumber ranged from 400 to 1800 cm‐1) that were taken at different depths. Profiles were defined by total ceramide content at skin depths of 5 and 10 μm, measured from the surface of the skin.

### 3.3 Transepidermal water loss (TEWL)

**Figure 3:**
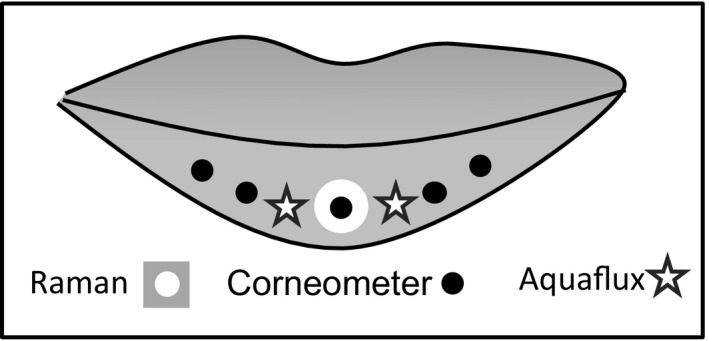
Measurement areas of the lower lip

Transepidermal water loss is a non‐invasive method to measure the barrier function of the skin and is regarded as a sensitive parameter to quantify skin barrier function. TEWL was measured with a closed chamber system using an Aquaflux AF200 (Biox Systems Ltd., London, UK) as described elsewhere.^24^ Water evaporation from the skin was measured by placing the cylindric chamber onto the lower lip. Two measurements per test area and assessment time were performed (Figure 3).

### 3.4 Stratum corneum hydration

The measurement of stratum corneum hydration was performed by the electrical capacitance method with a Corneometer CM 825 (Courage & Khazaka). Measurement principles are described elsewhere [24]. Five measurements per test area and assessment time were performed (Figure 3).

## 4 STATISTICS

Stratum corneum and stratum disjunctum thickness and water gradients were calculated from curves of total water content according to an appropriate non‐linear model. Additionally, total water contents within stratum corneum including stratum disjunctum and total water contents below the stratum corneum (total water content in deeper layer) were calculated as AUC (area under curves). Furthermore, the stratum corneum thicknesses were divided into three equally spaced areas. For each of these areas the AUC was calculated (Figure 2).

**Figure 4:**
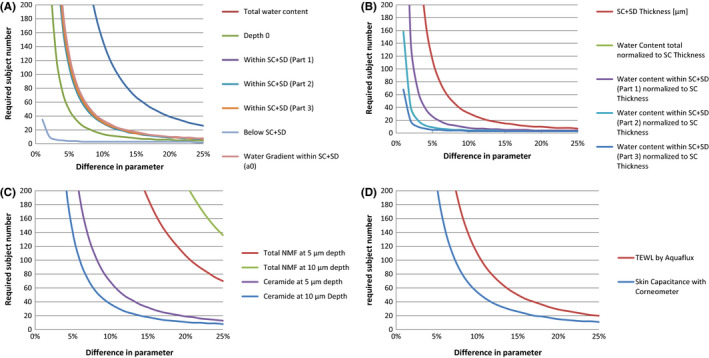
The actual required number of subjects for a statistically significant verification of a defined parameter

Results of water concentration profiles by Raman spectroscopy for all measured depths (0 to 24 μm) were summarized to mean concentration profiles (curves). Accordingly, ceramide and NMF in the two measured depths (5 and 10 μm) were summarized to mean concentrations over the repeated measurements per depth for both areas. For each instrumental parameter (TEWL and skin hydration) and each Raman water and ceramide/ NMF parameters, pairwise comparisons of treatments were performed with t test. Parameter distributions were assumed to be normal. For all test parameters means, standard deviations and confidence intervals were calculated. Plots with required sample size numbers for hypothetical percentual lip treatment effects were displayed (Figure 4).

The computation of the statistical data was carried out with commercially available statistics software (SAS for Windows).

## 5 RESULTS

Table 1 summarizes the data recorded for the three methods evaluated (Raman spectroscopy, Aquaflux, and corneometry) in the assessment of lip skin barrier. Mean values, standard deviations, and coefficient of variation for each parameter were reported alongside the calculated required percentage mean difference in the investigated population to detect a statistically significant change in the respective parameter (power ≥0.8).

**Table 1:** Parameters for lip barrier characterization within a population, their variability, and calculated necessary mean difference to achieve statistically significant distinction (power ≥0.8) of a given sample size

Parameter	Mean Value ± Standard deviation	Coefficient of Variation [%]	% Changes required for a significant treatment effect
01. Water content at depth 0 [%]	33.23 ± 4.05	12.2	7.64
02. Water content—AUC within SC + SD (Total)	925.21 ± 172.2	18.6	11.66
03. Water content—AUC within SC + SD (Part 1)	239.75 ± 45.06	18.8	11.77
04. Water content—AUC within SC + SD (Part 2)	308.80 ± 57.34	18.6	11.63
05. Water content—AUC within SC + SD (Part 3)	376.67 ± 72.43	19.2	12.05
06. Water content—AUC below SC + SD [SC + SD+15 µm]	974.26 ± 19.88	2.0	1.28
07. Water gradient within SC + SD (a0)	1.67 ± 0.34	20.4	12.57
08. Water gradient below SC + SD (a1)	0.22 ± 0.10	45.5	27.27
09. SC + SD thickness [µm]	19.49 ± 3.70	19.0	11.90
10. Water content—AUC within SC + SD (Total; Normalized on SC thickness [1/µm])	47.51 ± 2.12	4.5	2.80
11. Water content—AUC within SC + SD (Part 1; normalized on SC thickness [1/µm])	12.35 ± 1.06	8.6	5.43
12. Water content—AUC within SC + SD (Part 2; Normalized on SC thickness [1/µm])	15.86 ± 0.71	4.5	2.84
13. Water content—AUC within SC + SD (Part 3; Normalized on SC thickness [1/µm])	19.31 ± 0.56	2.9	1.86
14. Total NMFs [a.u.] at 5 µm depth	0.39 ± 0.29	74.4	46.15
15. Total NMFs [a.u.] at 10 µm depth	0.32 ± 0.33	103.1	63.49
16. Ceramide [a.u] at 5 µm depth	65.76 ± 19.09	29.0	17.75
17. Ceramide [a.u.] at 10 µm depth	43.26 ± 9.07	21.0	12.83
18. TEWL by Aquaflux [g/(m^2^h)]	51.44 ± 19.02	37.0	18.92
19. Skin capacitance with corneometer [a.u.]	40.49 ± 10.40	25.7	13.14

Note

Parameters 1‐13: n = 22; parameters 14‐17: n = 23; and parameters 18 and 19: n = 32.

As expected, water content assessed via Raman was lowest on the surface (parameter 1) and increased gradually (parameters 3‐5) until reaching a plateau (parameter 6). Accordingly, two water gradients were identified: a steep one within the SC (parameter 7) and a level one below SC (parameter 8). SC thickness was calculated at the point of intersect of these tangents (parameter 9). Furthermore, water content normalized to SC thickness (parameters 10‐13) was observed to increase with increasing depth. Both, NMF and ceramide content slightly decreased at greater depths (parameters 14‐17).

The required difference in parameter for significant distinction is a measure proportionate to the parameters' variability. From the three groups of Raman parameters assessed (water content and derived, NMF and ceramide) the largest variability was observed in the amount of NMF (parameters 14 and 15). The lowest variability was noted in the water content including increased skin depth (parameter 6). Notably, Raman parameters calculated by normalizing water content to stratum corneum thickness (parameters 10‐13) varied less than the values they have been calculated from.

Greater inter‐group variances were seen between the TEWL data and corneometry data (parameter 18 and 19), and these need to be taken into consideration when measuring these parameters. This is also corroborated by the variances and water gradient fluctuations seen in the Raman spectra (parameters 7 and 8). When evaluating the three methods used in this study, there was more precise less scattering of data with the Raman measurements as compared to TEWL and capacitance measurements.

For each parameter, the necessary number of subjects to detect a statistically significant change (power ≥0.8) was calculated. As expected, this number is proportionate to parameter variability and indirectly proportionate to the magnitude of change to be observed. In general, investigators seeking to verify smaller changes will therefore require the recruitment of higher subject numbers. This relationship for all parameters is illustrated in Figure 3.

## 6 DISCUSSION

Prior studies have demonstrated the combination of TEWL (open‐chamber) and corneometry^10^, ^11^ as tools for characterizing barrier function of lip skin. However they do not provide detailed information of the changes into the depth of the tissue that can be achieved with Raman spectroscopy. Skin barrier function, at the stratum corneum level, is normally evaluated using well‐established, non‐invasive biophysical methods such as TEWL and capacitance. However, these methods do not measure the skin's structure or composition. Often this will restrict skin barrier alteration causation. A more versatile technique, confocal Raman spectroscopy (CRS), can evaluate the structure and composition of the skin.

This study was therefore undertaken to establish consistency and validity of three combined methods for more purposeful lip skin barrier evaluation. To this end, for the first time, Raman spectroscopy in conjunction with well‐established methods of TEWL and capacitance measurements has been applied on the lip. In our study, we also found that a prerequisite for reproducible measurements was to fix the tongue with a dental device to prevent the involuntary reflex of lip moisturization.

Stable measurements enabled the assessment of a plethora of parameters relevant for in vivo characterization of the lip skin barrier. Our own unpublished results on forearm skin for example indicate that SC thickness tends to decrease in well‐kept skin and water content in the deepest part of the SC tends to increase with improved water barrier. Furthermore, we saw that an increased water gradient can be interpreted as a direct measure for skin barrier quality, as it signifies improved barrier properties achieved by thinner SC layers. The relevance of each parameter will vary in focus depending on the context of an investigation—ranging from fundamental skin physiology research to evaluation of cosmetic efficacy, as well as those parameters identified during the course of clinical studies.

In this study, the values obtained are not very different from those measurements undertaken on the volar forearm (Table 2) with the total water content of the lip mildly elevated as compared to forearm.^25^ SC thickness in lip skin is similar to the volar forearm and the legs. Interestingly, it is thicker than cheek skin.^26^ Not only was the SC thickness of the lip was found comparable to volar forearm skin, but the ceramide content was also quite similar^27^ (Tables 2 and 3). However, a distinctly higher rate of TEWL was observed on the lips than on the volar forearm.^28^ This finding is in agreement with the higher water content as assessed previously with Raman spectroscopy.^25^, ^29^ Therefore, when using CRS to obtain detailed information about the molecular composition of the skin, it is also possible to accurately measure SC thickness with the same device for an orientation in which skin layer molecules are found.

**Table 2:** Comparison of selected Raman parameters acquired on lip and volar forearm

Parameter	Lips skin		Volar forearm	
	N*	Mean values	N*	Mean values
Water content at depth 0 [%]	22	33	14	30^25^
Water content ‐AUC within SC + SD (Total)	22	925	14	854^25^
Total NMFs [a.u.]^a^	23	0.39	45	approx. 0.6‐0.8^27^
Ceramide [a.u]^a^	23	66	45	approx. 60^27^
Skin capacitance with corneometer [a.u.]	32	40	14	43^25^
TEWL by aquaflux [g/(m^2^h)]	32	51	16	14^28^

Note

N*, number of volunteers.

a For lip skin at 5 µm depth, for volar forearm skin at approximately 0‐8 µm depth.

**Table 3:** Comparison of the stratum corneum thickness of various body sites as measured in vivo with confocal Raman spectroscopy^26^

Body site	N*	Mean values	Standard deviation
Lips	22	19.5	3.7
Forearm	5	19.5	2.8
Palm	5	208.8	41.75
Cheek	5	12.8	1.51
Lower leg	5	22.4	2.2

Note

N*, number of volunteers.

Although measured parameter variability may have a number of physiological causes, this variability is an important indicator for establishing method reproducibility and hence highly relevant for future experimental design(s). For instance, the observed NMF variability could be explained as follows: Firstly, NMF is a collective term for several cutaneous water attracting components (eg, filaggrin degradation products, polycarboxylic acids, and urea) with different metabolic regulatory pathways. Thus the joint variability is not surprisingly larger. Secondly, filaggrin as a major component of NMF is formed gradually during the course of terminal keratinocyte differentiation. Hence, NMF forms a decreasing gradient actually increasing toward the SC surface. Relatively low amounts of NMF were found in lip SC. The values were approximately the half of what was found in volar forearm SC. Lower amounts of a compound necessarily correlate with higher variability as seen comparing the coefficients of the variability of NMF at 5 and 10 µm. Likewise, the high variability of the water gradient below the SC can be attributed to the low value of the gradient itself, illustrating the hydration capacity reaching a plateau value below the SC. Notably, the variability of water content normalized to SC thickness exhibits a lower variability than constituent values. This indicates a strong correlation between water content and SC thickness across the study population. Clearly, the water‐holding capacity of skin is a remarkably stable physiological parameter.

The required number of subjects for a statistically significant corroboration of a defined parameter change varies markedly between parameters. This number is proportionate to the variability of the parameter itself. Hence, determination of changes in water content and derived parameters (Figure 4A)—with the exception of deeper water—would require more subjects to significantly substantiate findings than actual changes in the water content normalized to SC thickness (Figure 4B). Significant changes in NMF would require the highest number of subjects for substantiation with ceramides ranging approximately in‐between (Figure 4C). Most Raman parameters require fewer subjects for statistically valid statements than long established TEWL and capacitance assessments (Figure 4D). However, an investigator must consider the physiology each parameter addresses and not just the numbers provided in Figure 4.

Importantly, parameters exhibiting a low inter‐individual variability and thus requiring low subject numbers for statistically sound statements may be particularly difficult to alter through external intervention by cosmetic ingredients or pharmaceutical drugs. Water content below the SC, for example, exhibits remarkably low variability (Table 1) resulting in a reduced necessity to corroborate even small changes in the parameter (Figure 4A). Nonetheless, this parameter is indicative of the primary SC function to maintain water content within. Changes to this parameter might be considerably more difficult to achieve than a significant reduction of TEWL by an emollient. Notwithstanding, these data are meant to serve as a tool to estimate necessary study populations after the magnitude of an effect on the parameter has been predicted as well is possible.

## 7 CONCLUSION

This in vivo confocal Raman spectroscopy led tri‐method approach in the evaluation of the lip barrier and skin lip hydration can be considered both relevant and valid. It offers a complimentary assessment of lip skin barrier functioning. For the first time in vivo, barrier‐relevant components of the lip (NMFs and Ceramide) are described. Inter‐subject variations also are considered for making statistical calculations required for such studies to ensure statistically significant distinction (power ≥0.8) between test groups for product evaluation. In vivo confocal Raman spectroscopy is able to detect differences at the molecular level and at a high spatial resolution, and these insights might provide a breakthrough in the evaluation of lip barrier function and developing solutions for lip care.
